# Inhibitory responses mediated by vagal nerve stimulation are diminished in stomachs of mice with reduced intramuscular interstitial cells of Cajal

**DOI:** 10.1038/srep44759

**Published:** 2017-03-20

**Authors:** Elizabeth A. H. Beckett, Kenton M. Sanders, Sean M. Ward

**Affiliations:** 1 Discipline of Physiology, Adelaide Medical School, University of Adelaide, Adelaide, SA, 5005, Australia; 2 Department of Physiology and Cell Biology, University of Nevada, Reno School of Medicine, Reno, NV, 89557, USA

## Abstract

Intramuscular interstitial cells of Cajal (ICC-IM) are closely associated with enteric motor nerve terminals and electrically coupled to smooth muscle cells within the gastric musculature. Previous studies investigating the role of ICC-IM in motor neurotransmission have used indiscriminate electric field stimulation of neural elements within the gastric wall. To determine the role of ICC-IM in transduction of vagally-mediated motor input to gastric muscles electrical and mechanical responses to selective electrical vagal stimulation (EVS) were recorded from gastric fundus and antral regions of wild type and *W/W*^*V*^ mice, which lack most ICC-IM. EVS evoked inhibitory junction potentials (IJPs) in wild type muscles that were attenuated or abolished by L-NNA. IJPs were rarely evoked in *W/W*^*V*^ muscles by EVS, and not affected by L-NNA. EVS evoked relaxation of wild type stomachs, but the predominant response of *W/W*^*V*^ stomachs was contraction. EVS applied after pre-contraction with bethanechol caused relaxation of wild type gastric tissues and these were inhibited by the nitric oxide synthase inhibitor L-NNA. Relaxation responses were of smaller amplitude in *W/W*^*V*^ muscles and L-NNA did not attenuate relaxation responses in *W/W*^*V*^ fundus muscles. These data suggest an important role for ICC-IM in vagally-mediated nitrergic relaxation in the proximal and distal stomach.

The vagus nerve is an autonomic extrinsic nerve that provides bi-directional connectivity between the stomach and the brainstem, modulates gastric motility, and mediates the gastric accommodation reflex^1^,^2^. The vagus contains 80–90% afferent nerve fibres that convey sensory information from the stomach to the central nervous system^3^,^4^ whilst parasympathetic efferents carry motor information to the gastric musculature from the dorsal motor nucleus^4^. Upon entering the gastric wall, efferent vagal nerve fibers form terminal arrays around enteric neurons in myenteric ganglia throughout the stomach, and vagal activity activates enteric motor neurons that innervate the smooth muscle layers and regulate motility^5^,^6^.

Previous studies have suggested a role for ICC-IM in both cholinergic excitatory and nitrergic inhibitory motor neurotransmission in the gastric fundus and antrum^7^,^8^,^9^,^10^,^11^, suggesting that ICC-IM are innervated, provide mechanisms of transduction of neural signals to the gastric musculature, and play a role in the gastric accommodation reflex. However, all previous studies of the role of ICC-IM have utilized short-duration pulses (0.3–0.5 ms) of electrical field stimulation (EFS) delivered at various frequencies to activate intrinsic neurons within gastric musculature. EFS of gastrointestinal tissues activates neural elements present within the stimulation field indiscriminately, and thus it is difficult to specifically assess the role of ICC-IM in vagally-mediated motor responses.

By preserving vagal branches entering the gastric musculature, we were able to selectively stimulate vagal fibres at a distance from the sites where post-junctional electrical and mechanical responses were recorded. We studied the importance of ICC-IM in mediating vagal motor responses by comparing responses in muscles from wild type and *W/W*^*V*^ mice, which have reduced populations of gastric ICC-IM^9^,^11^,^12^. This study demonstrates the importance of ICC-IM in nitrergic inhibitory responses that are initiated through vagal motor inputs to the stomach and are fundamental to gastric accommodation.

## Results

### Electrical post-junctional responses elicited by vagal stimulation

**Figure 1:**
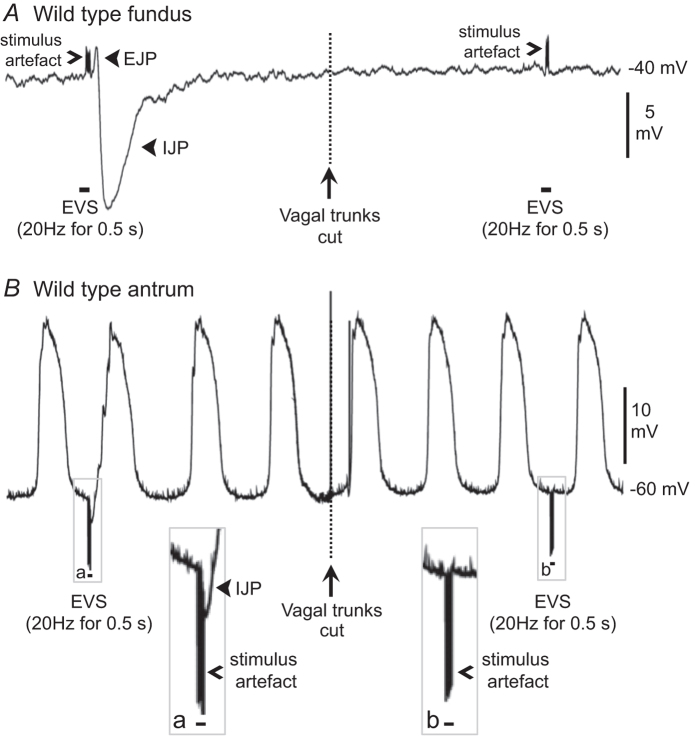
Typical post-junctional electrical responses elicited in wild type fundus and antrum by selective electrical stimulation of vagal trunks. (**A**) In wild type fundus multiple pulses of electrical stimulation applied to vagal trunks (EVS; 0.5 ms duration pulses delivered at 20 Hz for 0.5 s at black bars typically evoked a small excitatory junction potential (EJP) followed by a large amplitude inhibitory junction potential (IJP) that were both abolished by severing vagal trunks (at time point denoted by dashed line). Stimulus artefacts are indicated with an open arrowhead and junction potentials (EJP & IJP) are indicated with closed arrowheads. (**B**) Multiple pulse EVS (black bars) evoked small amplitude IJPs in the wild type antrum that were abolished by cutting vagal trunks (at time point denoted by dashed line). Responses to EVS prior to (**a**) and after (**b**) severing vagal branches demarcated by boxes are shown at x2 digital magnification in insets labeled **a** and **b** respectively. Stimulus artefacts are indicated with open arrowheads and IJP is indicated with closed arrowhead.

Short trains of electric vagal stimulation (EVS; 1–20 Hz applied for 0.5–1 s) elicited inhibitory junction potentials (IJPs) in the fundus and antral regions of wild type stomachs. In 3 of 6 fundus preparations, small amplitude (2–5 mV) excitatory junction potentials (EJPs) preceded IJPs, producing ‘biphasic’ excitatory and inhibitory responses. IJPs elicited by EVS were often composed of fast and slow components. Both components were blocked entirely by severing the vagal nerve trunks between the stimulating wires and gastric muscles (Fig. 1A), demonstrating that the IJPs were evoked by activation of the vagal trunks rather than by indiscriminate field stimulation through the solution bathing the muscles.

Cells impaled in antral muscles displayed ongoing electrical slow waves, as previously reported^13^. EVS, applied between slow waves, evoked IJPs that were followed closely by initiation of the next slow wave (i.e. phase advancement; Fig. 1B). EVS applied during the plateau phase of slow waves caused immediate repolarisation of the slow wave, effectively truncating or shortening the duration of the slow wave (data not shown). Cutting vagal trunks also abolished post-junctional responses to EVS in the antrum (Fig. 1B).

**Figure 2:**
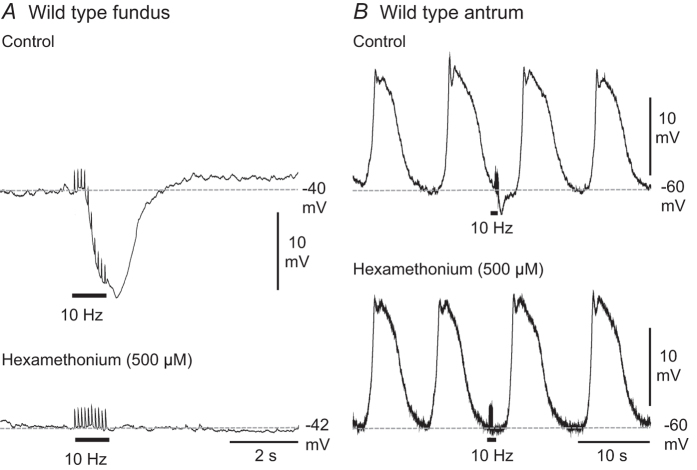
The nicotinic receptor blocker hexamethonium abolished post-junctional electrical responses to EVS in wild type fundus and antrum. (**A**) In wild type fundus, EVS (0.5 ms duration pulses delivered at 10 Hz for 1 s) elicited inhibitory junction potentials that were abolished by 500 μM hexamethonium. (**B**) In wild type antrum, small amplitude IJPs typically evoked by multiple pulse EVS in control conditions were abolished by hexamethonium.

We tested whether responses to EVS were mediated through nicotinic neurotransmission. Hexamethonium (500 μM) abolished post-junctional responses of fundus and antrum muscles to EVS (Fig. 2A,B; n = 4). These data suggest that synaptic transmission from vagal efferents to myenteric motor neurons was mediated through nicotinic receptors, as previously documented^14^.

**Figure 3:**
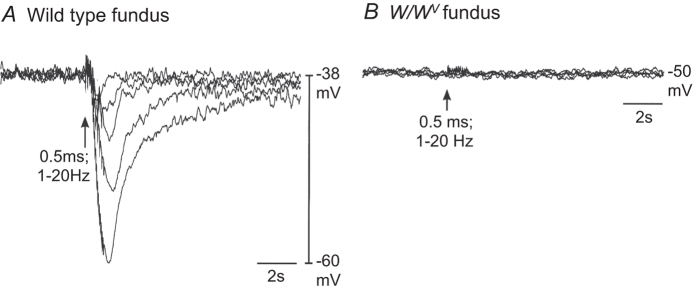
Post-junctional electrical responses to vagal stimulation in wild type and *W/W*^*V*^ fundus. (**A**) In wild type fundus, vagal stimulation (single pulse; 0.5 ms duration, and trains consisting of pulses applied at 3, 5, 10 and 20 Hz for 1 s) elicited inhibitory junction potentials, with graded increases in amplitude as frequency of stimulation was increased. (**B**) Spontaneous unitary potential activity (present in wild type muscles) was reduced in *W/W*^*V*^ fundus muscles and vagal stimulation failed to elicit post-junctional electrical responses in 4 of 6 preparations.

IJPs evoked by EVS increased in amplitude in wild type fundus muscles as a function of the frequency of stimulation (i.e. 1–20 Hz; Fig. 3A). There was a marked reduction in post-junctional responses to EVS in fundus muscles of *W/W*^*V*^ mice. In 4 of 6 *W/W*^*V*^ fundus muscles EVS failed to elicit any post-junctional responses (Fig. 3B) whereas in the remaining two *W/W*^*V*^ muscles EVS evoked IJPs. Excitatory junction potentials were not elicited by EVS in any of the 6 *W/W*^*V*^ fundus preparations.

**Figure 4:**
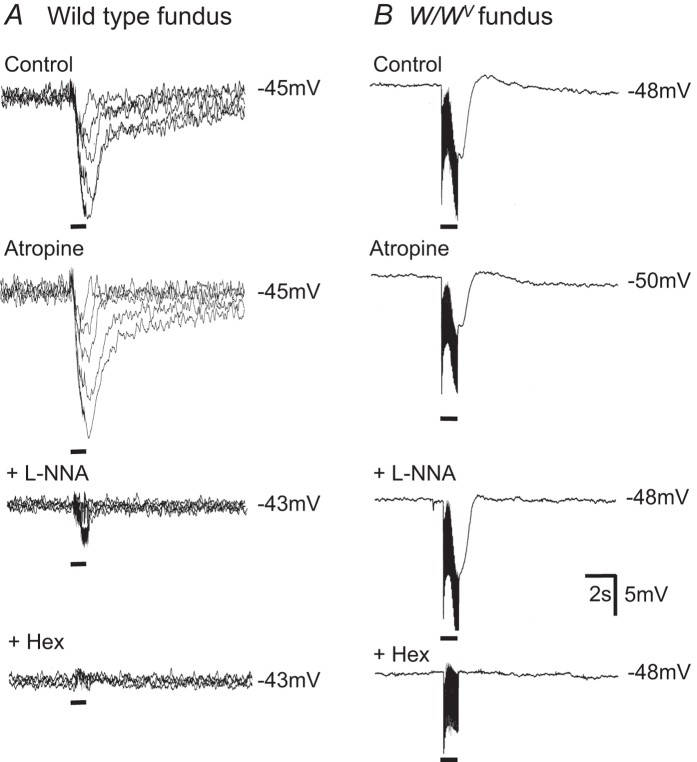
Intracellular electrical recordings reveal differences in pharmacological sensitivity of IJPs elicited in wild type and *W/W*^*V*^ fundus. (**A**) Vagal stimulation (single pulse, 3, 5, 10 and 20 Hz; delivered at bars for 1 s) elicited IJPs with graded amplitudes in wild type fundus. Traces obtained at each stimulation frequency have been superimposed. IJPs obtained under control conditions were potentiated by 1 μM atropine and attenuated by 100 μM L-NNA. Residual vagal inhibitory responses in the presence of L-NNA were abolished by the nicotinic antagonist hexamethonium (Hex; 500 μM). (**B**) In 2 of 6 *W/W*^*V*^ fundus preparations, vagal stimulation (20 Hz; delivered at black bars) elicited IJPs that were largely unaltered by atropine and L-NNA but abolished by hexamethonium.

The nature of the neurotransmitters involved in responses to EVS was investigated using drugs to eliminate specific components. Figure 4A illustrates electrical responses of wild type fundus muscles to graded frequencies of EVS (i.e. 1, 3, 5, 10 & 20 Hz) in control conditions, and following cumulative addition of atropine, L-NNA and hexamethonium. Atropine (1 μM) abolished any small EJPs and increased IJP amplitudes. In the continued presence of atropine, L-NNA (100 μM) caused a significant reduction in IJPs at all frequencies. Small IJPs remaining in the presence of L-NNA were abolished by hexamethonium (500 μM; Fig. 4A). Atropine and L-NNA had little or no effect on the IJPs elicited in the two *W/W*^*V*^ fundus muscles in which IJPs were observed (Fig. 4B). Responses in these muscles were abolished by hexamethonium (Fig. 4B)

### Mechanical responses of gastric fundus elicited by vagal stimulation

**Figure 5:**
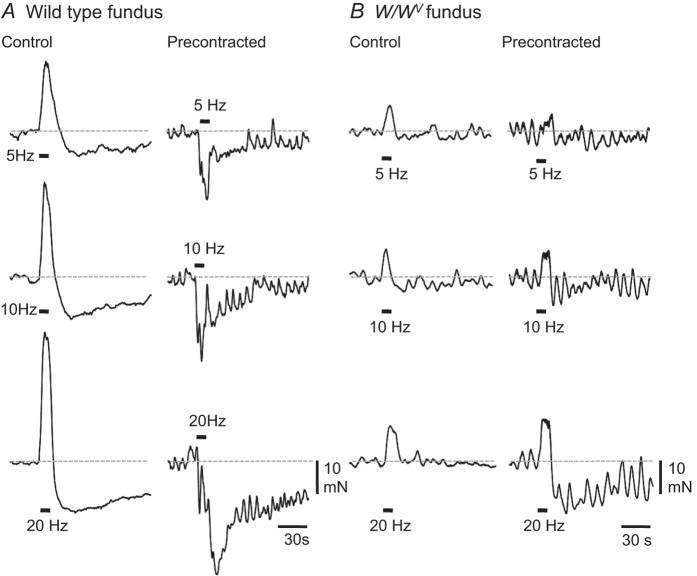
Typical mechanical responses elicited in wild type and *W/W*^*V*^ fundus by EVS. (**A**) biphasic contraction-relaxation responses evoked by 5, 10 and 20 Hz EVS in wild type fundus in control conditions; when precontracted with bethanechol (3 μM) EVS elicited graded fundal relaxations, with the amplitude of relaxation being dependent upon the frequency of vagal stimulation applied. (**B**) In control conditions vagal stimulation commonly produced contraction of *W/W*^*V*^ fundus; in the presence of bethanechol, contraction responses persisted.

**Figure 6:**
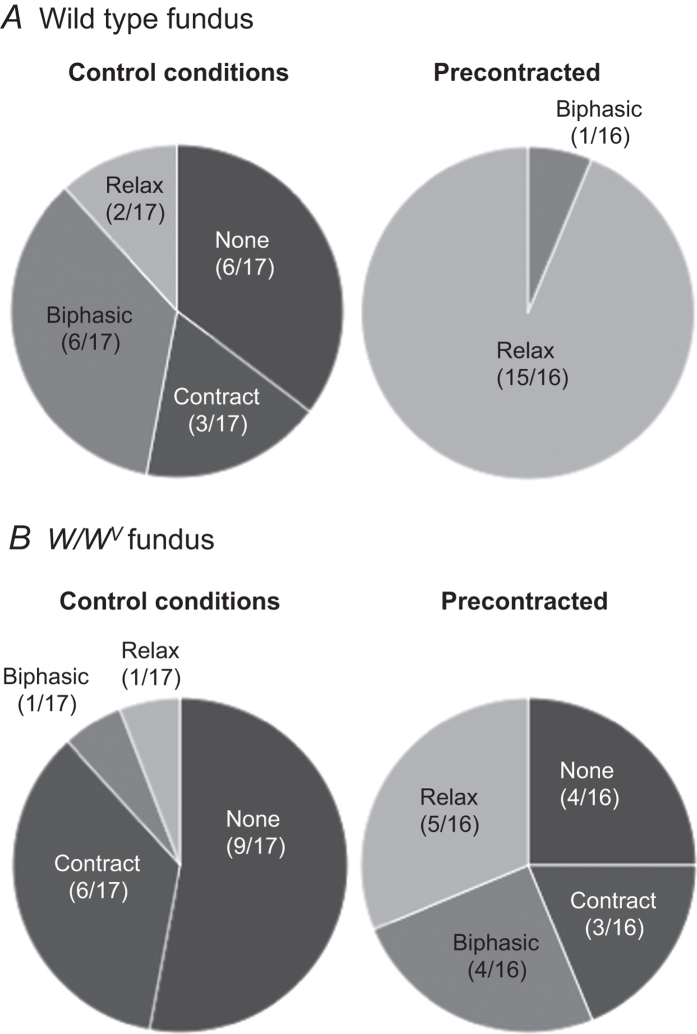
Mechanical response profiles of wild type and *W/W*^*V*^ fundus to 20 Hz vagal stimulation. (**A**) In control conditions, vagal stimulation produced variable mechanical responses in wild type fundus with biphasic responses (comprised of contraction followed by relaxation) being evoked in 6 of 17 preparations; in bethanechol (3 μM) 20 Hz EVS elicited relaxations in over 90% (15 of 16) of wild type fundus. (**B**) Of the 8 *W/W*^*V*^ fundus preparations that responded mechanically to vagal stimulation in control conditions, 6 exclusively contracted; in bethanechol, EVS elicited a variety of *W/W*^*V*^ fundus mechanical responses, with only 5 of 16 exhibiting relaxations at 20 Hz stimulation. Contract - exclusively contracted; Relax - exclusively relaxed; Biphasic - mechanical responses comprised of contraction followed by relaxation; None - no detectable mechanical response upon vagal stimulation. (n = 17 for each group in control conditions; n = 16 for each group in bethanechol).

Mechanical responses of wild type fundus preparations to EVS (5, 10 & 20 Hz; 10 s trains) were variable. In 6 of 17 wild type preparations EVS evoked frequency-dependent biphasic mechanical responses that consisted of an initial rapid contraction followed by a sustained relaxation (an example of this response type is shown in Fig. 5A). In 3 wild type fundus muscles only contraction occurred in response to EVS, and in 2 only relaxation responses were observed. In the remaining 6 wild type fundus muscles EVS failed to elicit detectable mechanical responses. Mechanical responses of wild type fundus muscles to 20 Hz EVS are summarised in Fig 6A. To better resolve relaxation responses, gastric tissues were precontracted with bethanechol (3 μM). Under these conditions significant resolution of the relaxation responses of fundus muscles to EVS was accomplished, and more than 90% of muscles (15 of 16) displayed relaxation responses to EVS. An example is shown in Fig. 5A, and the mechanical response pattern to 20 Hz EVS in pre-contracted wild type fundus muscles is shown in Fig. 6A.

Under control conditions and in muscles pre-contracted with bethanechol, EVS-evoked response profiles of *W/W*^*V*^ fundus muscles differed from wild type responses. In control conditions 50% of *W/W*^*V*^ stomachs (9 of 17) EVS failed to produce mechanical responses at any stimulation frequency tested. Exclusively contractile responses were elicited by EVS in 7 of 17 *W/W*^*V*^ fundus muscles (an example is shown Fig. 5B). The secondary long-lasting relaxation component, prominent in many wild type muscles, was absent in most *W/W*^*V*^ muscles (Fig. 5B). In a single *W/W*^*V*^ fundus preparation a biphasic ‘contraction-relaxation’ response to EVS was evident whilst in another EVS elicited a small relaxation response but no initial contractile response was observed.

After pre-contraction with bethanechol EVS produced variable responses in *W/W*^*V*^ fundus muscles. In contrast to wild type fundus muscles, contractions were elicited in 7 of 16 pre-contracted *W/W*^*V*^ fundus: 4 of these muscles displayed small secondary relaxation responses (example provided in Fig. 5B), and 3 displayed only contractile responses. In 5 of 16 muscles small relaxation responses were evoked and in a 4 pre-contracted *W/W*^*V*^ fundus muscles mechanical responses to EVS were not detected. *W/W*^*V*^ mechanical response patterns to 20 Hz EVS in control and pre-contracted conditions are summarised in Fig. 6B.

**Figure 7:**
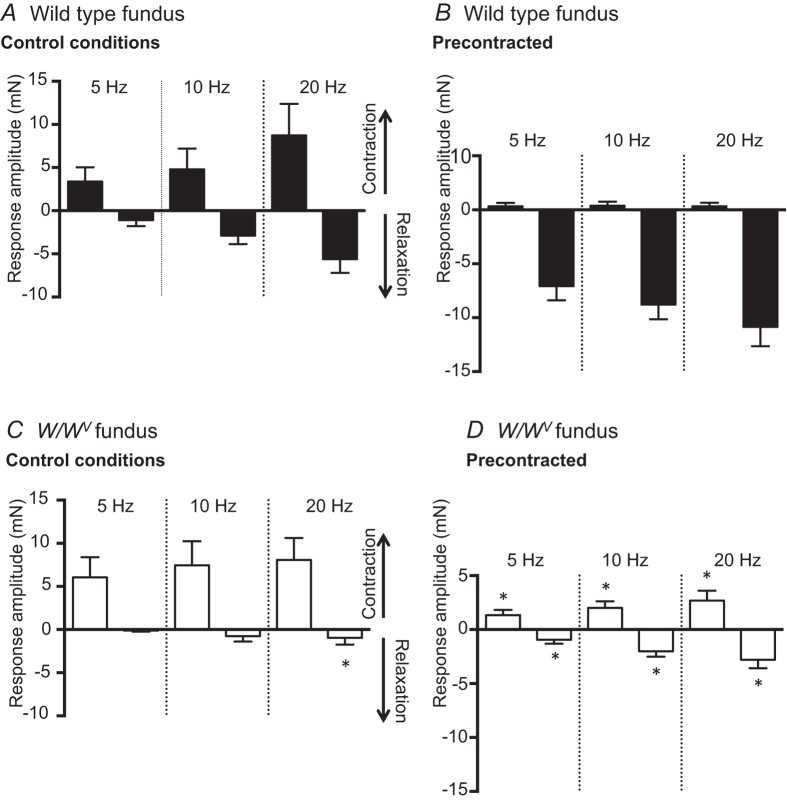
Summarised relaxation and contraction amplitudes evoked in wild type and *W/W*^*V*^ fundus by vagal stimulation (5, 10 and 20 Hz). (**A**) Contractions followed by relaxations were evoked by vagal stimulation of wild type fundus muscles under control conditions. Pre-contraction of fundus muscles with bethanechol altered the vagal stimulation response profile to almost exclusive relaxation. (**B**) *W/W*^*V*^ fundus muscle contractions elicited by vagal stimulation did not differ significantly from wild type muscles however relaxations evoked by 20 Hz vagal stimulation were significantly attenuated compared to wild type controls. In bethanechol, *W/W*^*V*^ fundus contractions were larger and relaxation amplitudes reduced compared to pre-contracted wild type fundus. (*denotes p ≤ 0.05 compared to same condition in wild type; n = 16 animals for each group).

Responses to EVS are summarized in Fig. 7 and show that the amplitudes of contractions and relaxations were frequency dependent and EVS produced significantly larger relaxation responses in wild type than *W/W*^*V*^ muscles. Pre-contraction of wild type fundus with bethanechol altered the response pattern to EVS, causing responses to change to dominant relaxation responses (compare Fig. 7A with B). The mean amplitude of contractions evoked in *W/W*^*V*^ muscles by EVS did not differ significantly as a function of stimulus frequency, and relaxation responses were smaller compared to those evoked in wild type muscles (Fig. 7C). In pre-contracted *W/W*^*V*^ muscles, small contraction components persisted whilst the mean amplitudes of relaxations were smaller in amplitude than those elicited in wild type muscles under the same conditions (compare Fig. 7B with D).

**Figure 8:**
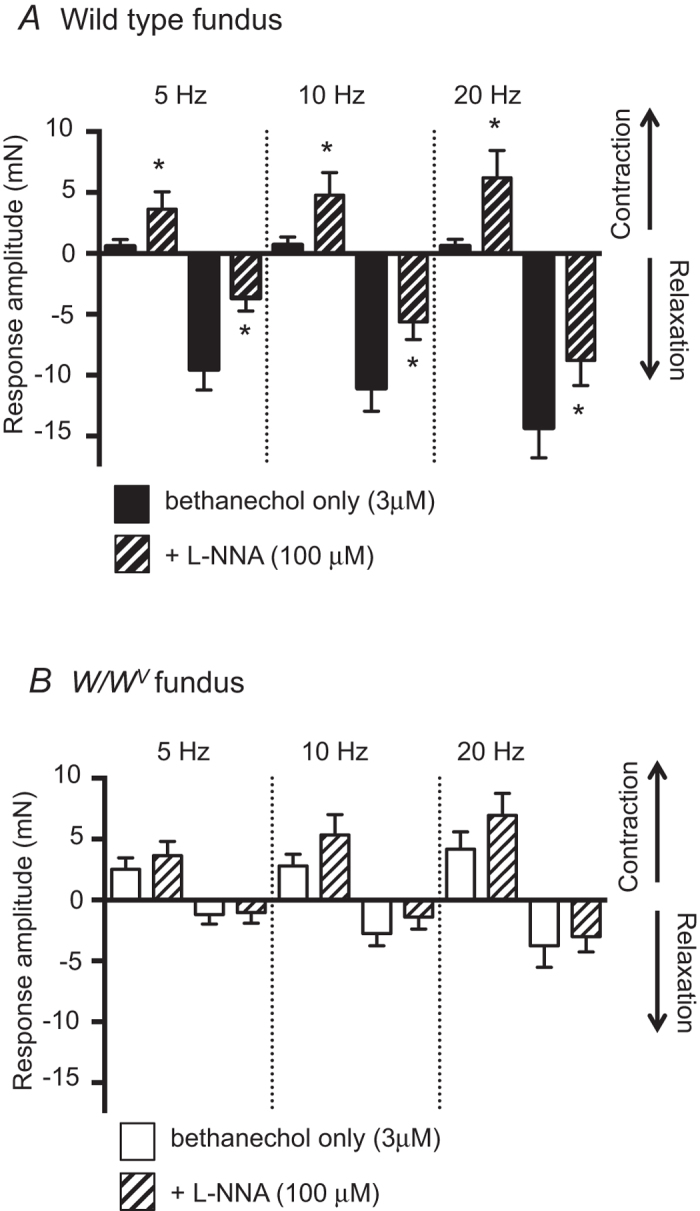
Effect of nitric oxide synthase inhibition on vagally evoked mechanical responses of pre-contracted wild type and *W/W*^*V*^ fundus muscles. (**A**) L-NNA (100μM) increased EVS evoked contractions and reduced relaxation amplitudes in pre-contracted wild type fundus (n = 6) [*denotes p ≤ 0.05 compared to responses in bethanechol only]. (**B**) L-NNA did not significantly alter amplitudes of either contractions nor relaxations elicited in *W/W*^*V*^ fundus by vagal stimulation (n = 6).

L-NNA significantly increased contractile responses evoked by EVS in wild type pre-contracted fundus muscles (n = 6; Fig. 8A). In contrast, L-NNA did not significantly affect the amplitudes of contractions or relaxations evoked in pre-contracted *W/W*^*V*^ fundus muscles (n = 6; Fig. 8B).

### Mechanical responses of antral muscles evoked by EVS

**Figure 9:**
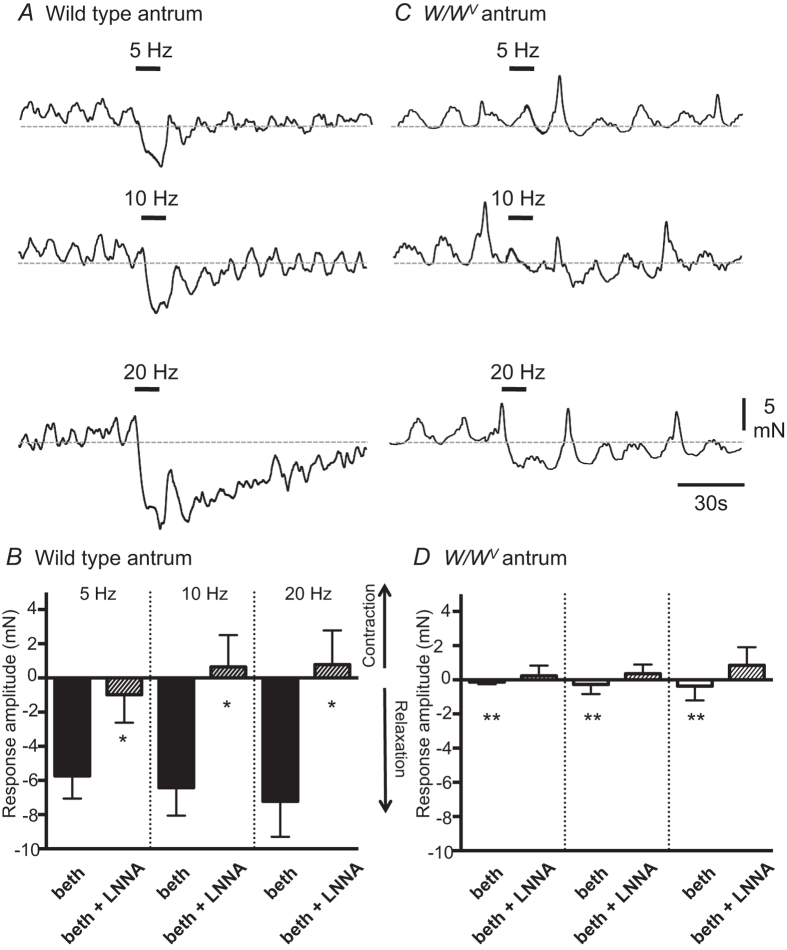
Mechanical responses elicited in pre-contracted wild type and *W/W*^*V*^ antrum by vagal stimulation. (**A**) In bethanechol (3 μM), EVS (5, 10 & 20 Hz delivered at bars as indicated) elicited graded relaxations of wild type antrum, with relaxation amplitude increasing as the frequency of vagal stimulation increased. (**B**) Summarised relaxation and contraction amplitudes evoked in wild type antrums by electrical vagal stimulation (EVS; 5, 10 and 20 Hz) in bethanechol (3 μM) only (beth) and in bethanechol and L-NNA (100 μM) (beth + LNNA) (n = 8; *denotes a significant difference (p < 0.05) between response amplitudes elicited in beth + L-NNA compared to beth only). (**C**) EVS produced relaxations of pre-contracted *W/W*^*V*^ antrums, but these were typically of smaller amplitude than those elicited in wild type antrum muscles. (**D**) Summarised relaxation and contraction amplitudes evoked in *W/W*^*V*^ antrum by EVS (5, 10 and 20 Hz) in bethanechol (3 μM) only (beth) and after L-NNA (100 μM) (beth + LNNA) (n = 7; **denotes a significant difference in response amplitudes (p < 0.001) elicited in *W/W*^*V*^ antrums compared to same condition in wild type antrums).

Mechanical responses of the gastric antrum to EVS were also examined in wild type and *W/W*^*V*^ stomach preparations. Antral muscles displayed very little basal tone, so these experiments were performed after pre-contraction with bethanachol. In the presence of bethanechol, EVS consistently caused frequency-dependent relaxations of wild type antrum that were significantly reduced by L-NNA (100 μM) (Fig. 9A,B; n = 8). In contrast, small EVS-evoked relaxations (1–3 mN) were only produced in 2 of 7 *W/W*^*V*^ antrum preparations (example shown in Fig. 9C) – in one, these small relaxation responses were abolished by L-NNA and in the other relaxations were reduced by approximately 50%. In 4 *W/W*^*V*^ preparations no mechanical responses could be resolved following EVS either in bethanechol alone or after the addition of L-NNA. In one *W/W*^*V*^ antrum small (2–3 mN) EVS-evoked contractions were evoked by higher frequency EVS (10 and 20 Hz) and these were potentiated by L-NNA. Summarised *W/W*^*V*^ antrum data is provided in Fig. 9D.

## Discussion

This study shows that vagal inhibitory responses, which are important for physiological responses, such as gastric accommodation, are linked to contractile behaviour, in part, through ICC-IM. There has been controversy about the role of ICC in neural responses in the stomach^10^,^11^,^12^,^13^,^15^,^16^, and we wondered whether part of this controversy stemmed from the use of indiscriminant electrical field stimulation that activates, possibly to varying degrees in different experimental conditions, all neural elements within the gastric musculature. Efferent vagal output, originating in the dorsal motor nucleus of the vagus nerve, regulates gastric motility responses, such as gastric accommodation, and may be focused upon specialized pathways of enteric motor neurons. We found that hexamethonium-sensitive inhibitory junction potentials elicited by EVS were largely absent in *W/W*^*V*^ mice with reduced ICC-IM. IJPs were evoked in a few *W/W*^*V*^ muscles, but these responses were insensitive to L-NNA. Contractile responses were also altered in *W/W*^*V*^ in that excitatory responses were of similar magnitude, but inhibitory responses were greatly reduced in magnitude. This was particularly true in pre-contracted muscles where relaxation was the dominant response in wild type fundus muscles and excitatory and inhibitory responses were evoked in *W/W*^*V*^ muscles. L-NNA enhanced contractile effects and blocked approximately 50% of the relaxation responses in wild type muscles but had little or no effect on the contractile responses of *W/W*^*V*^ muscles. Similar effects were noted on the contractile responses of antral muscles. Taken together, these experiments suggest involvement of ICC-IM in the nitrergic component of vagal motor inputs to the proximal and distal stomach.

The current study supports a previous report showing that nitrergic IJPs are largely abolished in fundus muscles of *W/W*^*V*^ mice^9^. Others have argued that nitrergic inhibitory responses are intact and normal in the lower oesophageal sphincter of *W/W*^*V*^ mice, and suggest that there is no obligatory role for ICC-IM in nitrergic regulation within gastrointestinal tissues^17^,^18^. In a re-evaluation of this question we found that the lesion in ICC-IM varies to some extent in different animals. When ICC-IM were greatly reduced, nitrergic responses were greatly reduced, but when clusters of ICC-IM remained, nitrergic responses were observed^12^. A recent study using cell-specific inducible Cre recombinase (iCre) to knock down soluble guanylate cyclase (sGC) found that nitrergic responses were affected only to a minor extent when the lesion in sGC was directed at ICC and only partially decreased when sGC was knocked down in smooth muscle cells^19^. Reduction in sGC in both cell-types abolished nitrergic relaxation, and these authors suggested that nitrergic responses were mediated jointly by smooth muscle cells and ICC^19^. While cell-specific manipulation of gene expression is an elegant approach, we have questioned whether the knockdown of sGC in ICC is quantitative using the iCre approach and whether remaining sGC could persist in mediating nitrergic responses in these mice^20^. Alternative explanations for the discordant observations of the present study and others might include remodeling of smooth muscle cells in *W/W*^*V*^ that reduce nitrergic responses in these cells.

It is possible that studies performed with electrical field stimulation do not discriminate the subtleties of selective pathways innervated and activated during vagal reflexes. Field stimulation of muscles activates neural elements indiscriminately, while EVS activates hexamethonium-sensitive pathways that are activated by vagal efferent input. Perhaps post-ganglionic motor neurons activated by vagal inputs are directed more specifically at ICC-IM. Something akin to this idea has been observed in studies of the lower esophageal sphincter (LES), using experiments in which iCre expression was directed toward either ICC or SMC^21^. In these studies swallow-induced changes in LES tone were dependent prominently upon ICC, while responses to field stimulation appeared to be mediated by both smooth muscle cells and ICC. Relaxation of the LES in swallowing is another reflex mediated by vagal input.

An earlier report provided evidence for a role of ICC-IM in the transduction of cholinergic excitatory responses evoked by non-discriminate electrical field stimulation^11^; a concept which has been debated^15^,^17^. In this study the excitatory phase of mechanical responses to selective vagal stimulation persisted in *W/W*^*V*^ gastric muscles. Indeed EVS-evoked contractions in *W/W*^*V*^ fundus were slightly but significantly larger than wild type controls under some conditions (see Figs 8 and 9). It is likely that the reduced nitrergic relaxations, evident in *W/W*^*V*^ muscles, is at least partially responsible for this apparent increase in contraction amplitude. i.e. when L-NNA was applied to wild type muscles an increase in contractile response was observed, but this was not seen in *W/W*^*V*^ animals (Fig. 9). Previous studies have reported changes in Ca^2+^ sensitization mechanisms in *W/W*^*V*^ muscles^22^ and these changes might also contribute to preservation of cholinergic excitatory responses in muscles lacking most ICC-IM. In wild type mice where ICC and close synaptic contacts between ICC and enteric motor nerve varicosities are intact only the pathway for Ca^2+^ sensitization mediated by CPI-17 was activated. When muscarinic stimuli were applied to the solution bathing muscles or when most ICC were lost in *W/W*^*V*^ muscles, Ca^2+^ sensitization pathways utilizing phosphorylation of CPI-17 and MYPT1 were both activated. Recruitment of an additional Ca^2+^ sensitization mechanism(s) could help preserve excitatory cholinergic responses when ICC-IM are lost or damaged.

In summary, our data suggest an important role for ICC-IM in nitrergic inhibitory responses in the stomach. There were also changes in the excitatory responses (i.e. no excitatory junction potentials were elicited in *W/W*^*V*^ gastric muscles), however contractile responses were better preserved in *W/W*^*V*^ muscles than were inhibitory relaxation responses. Inhibition of contraction is extremely important in the gastric accommodation reflex, as it increases gastric wall compliance to accommodate the increase in volume when food is ingested. Gastric accommodation is largely a vagally-mediated reflex, so our results would predict that loss of ICC-IM could compromise this motility behaviour and might lead to premature feelings of fullness or bloating when ICC-IM are lost or damaged.

## Methods

### Animals and tissue preparation

Wild type (*Kit*^+^/*Kit*^+^) and *W/W*^*V*^ (*Kit*^*W*^/*Kit*^*W*-*v*^) mice were bred within the Laboratory Animal Services facility at the University of Adelaide from female *WB/ReJ*-*Kit*^*W*^/*Kit*^+^ (*W*/+) and male *C57BL/6J*-*KitW*^*V*^/*Kit*^+^ (*W*^*V*^/+) mice, originally obtained from The Jackson Laboratory (Bar Harbor, ME, USA). All experimental procedures were in accordance with the Australian code for the care and use of animals for scientific purposes and were approved by the University of Adelaide Animal Ethics Committee.

**Figure 10:**
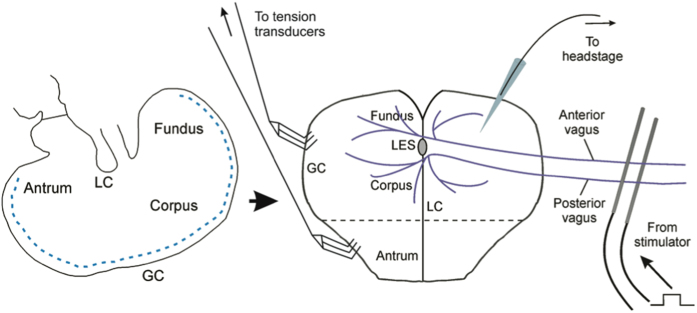
Experimental setup used to record electrical and mechanical responses of gastric fundus and antral regions to electrical vagal stimulation. Stomachs were opened along greater curvature (curved dashed line) with oesophageal vagal trunks intact. Platinum stimulating wires were positioned above and below vagal trunks and used to deliver multiple pulses of electrical vagal stimulation (EVS; 0.5 ms pulse duration; 5–20 Hz; 1–10 s trains). Mechanical responses to EVS were recorded from fundus and antral regions (i.e. above and below straight dashed line respectively) via small stainless steel rakes attached to isometric tension transducers with suture thread.

Wild type and *W/W*^*V*^ animals of either sex, aged between 6 and 12 months, were anaesthetised by isoflurane inhalation and culled via cervical dislocation. Stomachs, together with oesophagus and intact anterior and posterior vagal trunks were removed via a thoracoabdominal incision. Stomachs were opened along greater curvature (dashed line, Fig. 10), and gastric contents flushed away, taking care to ensure that oesophagus and associated vagal trunks remained intact and free from damage. Vagal trunks were carefully isolated from the proximal oesophagus to enable platinum stimulating wires to be positioned above and below vagal trunks, approximately 15 mm from the lower oesophageal sphincter (Fig. 10). Stomach preparations were constantly perfused with oxygenated Krebs solution (see *Solutions and drugs* for composition) maintained at 36 ± 1 °C.

Electrical vagal stimulation (EVS; 0.5 ms pulse duration; 5–20 Hz; 1–10 s trains) was delivered via an electrical stimulator (Grass S48; Grass Technologies, WI, USA) connected to platinum electrodes positioned above and below vagal trunks. For intracellular electrophysiology experiments, membrane potential was monitored by impalement of smooth muscle cells with high-resistance (80–120 MΩ) electrodes filled with 3 M KCl. Membrane potentials were amplified by a standard microelectrode amplifier (Axoclamp-2B, Molecular Devices, CA, USA) and analogue-to-digital conversion was performed (Digidata 1322a, Molecular Devices, CA, USA) to allow recording on a PC running Axoclamp 9.0 software. Mechanical activities of gastric sheets were recorded via small stainless steel rakes attached to high sensitivity isometric tension transducers (AD Instruments MLT0202; Bella Vista, NSW, Australia) with silk suture thread. Mechanical responses were amplified using a quad bridge amp (AD Instruments ML224; Bella Vista, NSW, Australia), before being converted to digital signals (Powerlab 4/35 data acquisition device, AD Instruments, Bella Vista, NSW, Australia) and recorded for later analysis on a PC running LabChart 7 software (AD Instruments; Bella Vista, NSW, Australia). Figure 10 illustrates the preparation used to record electrical and mechanical responses to selective stimulation of vagal nerve trunks.

### Solutions and drugs

Krebs solution (used in both mechanical and intracellular electrophysiology experiments) contained in mM: NaCl 118.0; KCl 4.75; NaHCO_3_ 25.0; glucose 11.0; MgSO_4_ 1.2; NaH_2_PO_4_ 1.0; CaCl_2_ 2.5. Krebs pH was 7.3–7.4 when bubbled with 95% O_2_–5% CO_2_ and maintained at 36 ± 1 °C.

Hexamethonium chloride; atropine sulphate; N-Nitro-L-arginine (L-NNA) and bethanechol chloride were obtained from Sigma-Aldrich (Merck Millipore, St. Louis, MO, USA). Each drug was initially dissolved in water to produce a stock solution of 10^−2^ M and diluted in Krebs solution to the final concentrations stated in the text.

### Data Analysis

Data are expressed as means ± standard errors of the mean (SEM). The n values reported in the text refer to the number of stomach preparations used. Figures were constructed from digitized data traces using Microsoft Powerpoint 2011 (Microsoft Corporation) and Adobe Illustrator CS6 (Adobe Systems Incorporated). Prism 6.0 (GraphPad Software Incorporated) was used to construct summary data charts and also to run statistical tests. Student’s t-test or one-way ANOVA followed by Tukey’s multiple comparison post-test were used to determine if differences were statistically significant. P values < 0.05 were considered to represent significant changes.

## Additional Information

**How to cite this article:** Beckett, E. A. H. *et al*. Inhibitory responses mediated by vagal nerve stimulation are diminished in stomachs of mice with reduced intramuscular interstitial cells of Cajal. *Sci. Rep.* **7**, 44759; doi: 10.1038/srep44759 (2017).

**Publisher's note:** Springer Nature remains neutral with regard to jurisdictional claims in published maps and institutional affiliations.
